# Mitochondrial alarmins are tissue mediators of ventilator-induced lung injury and ARDS

**DOI:** 10.1371/journal.pone.0225468

**Published:** 2019-11-22

**Authors:** Serge Grazioli, Irène Dunn-Siegrist, Laure-Anne Pauchard, Mathieu Blot, Pierre-Emmanuel Charles, Jérôme Pugin

**Affiliations:** 1 Intensive Care Laboratory, Department of Microbiology and Molecular Medicine, University Hospitals of Geneva & Faculty of Medicine, Genève, Switzerland; 2 Department of Pediatrics, Division of Neonatal and Pediatric Intensive Care, University Hospital of Geneva, Genève, Switzerland; 3 Intensive Care Unit, University Hospital of Dijon, Dijon, France; 4 U.M.R. 1231, I.N.S.E.R.M, Burgundy University, Dijon, France; 5 Department of Infectious Diseases, University Hospital of Dijon, Dijon, France; Forschungszentrum Borstel Leibniz-Zentrum fur Medizin und Biowissenschaften, GERMANY

## Abstract

**Rationale:**

Endogenous tissue mediators inducing lung inflammation in the context of ventilator-induced lung injury (VILI) and acute respiratory distress syndrome (ARDS) are ill-defined.

**Objectives:**

To test whether mitochondrial alarmins are released during VILI, and are associated with lung inflammation.

**Methods:**

Release of mitochondrial DNA, adenosine triphosphate (ATP), and formyl-Met-Leu-Phe (fMLP) peptide-dependent neutrophil chemotaxis were measured in conditioned supernatants from human alveolar type II-like (A549) epithelial cells submitted to cyclic stretch *in vitro*. Similar measurements were performed in bronchoalveolar lavage fluids from rabbits submitted to an injurious ventilatory regimen, and from patients with ARDS.

**Measurements and main results:**

Mitochondrial DNA was released by A549 cells during cell stretching, and was found elevated in BAL fluids from rabbits during VILI, and from ARDS patients. Cyclic stretch-induced interleukin-8 (IL-8) of A549 cells could be inhibited by Toll-like receptor 9 (TLR9) blockade. ATP concentrations were increased in conditioned supernatants from A549 cells, and in rabbit BAL fluids during VILI. Neutrophil chemotaxis induced by A549 cells conditioned supernatants was essentially dependent on fMLP rather than IL-8. A synergy between cyclic stretch-induced alarmins and lipopolysaccharide (LPS) was found in monocyte-derived macrophages in the production of IL-1ß.

**Conclusions:**

Mitochondrial alarmins are released during cyclic stretch of human epithelial cells, as well as in BAL fluids from rabbits ventilated with an injurious ventilatory regimen, and found in BAL fluids from ARDS patients, particularly in those with high alveolar inflammation. These alarmins are likely to represent the proximal endogenous mediators of VILI and ARDS, released by injured pulmonary cells.

## Introduction

Mechanical ventilation has been lifesaving in many patients with respiratory failure since its introduction several decades ago. It has also been associated with lung damage due to cyclic stretch imposed by positive pressure mechanical ventilation, particularly when the lung is injured or infected. This phenomenon is nowadays known as ventilator-induced lung injury (VILI) [1, 2]. It is also now widely accepted that ventilatory strategies aimed at decreasing airway overstretching are associated with better outcome in patients with and without acute respiratory distress syndrome (ARDS) [3, 4].

An important part of VILI is due to lower airway inflammation mediated by pro-inflammatory cytokines, chemokines, and blood neutrophils recruited to the airways [5–7]. In *vitro* experiments with isolated lung cells [8, 9] and animal studies with intact lungs [10–12] could demonstrate that cyclic stretch *per se* induced pro-inflammatory mediators such as monocyte chemoattractant protein-1 (MCP-1) and interleukin-8 (IL-8), prototypical chemokines for myeloid cells. Although it could be shown that cyclic stretch induces *de* novo transcription of IL-8 gene [13], it remains unclear whether this was a direct transcriptional effect of the mechanical strain or a secondary effect of endogenous mediator(s) released by lung cells injured by cyclic stretch.

Mechanical ventilation and cyclic stretch of lung cells have been shown to induce cell membrane breaks with spillover of intracellular cell content [14, 15]. Recent evidence suggests that injured cells release alarmins (also called damage-associated molecular patterns, DAMPs) originating from mitochondria [16–18], and that these alarmins may play a role in mediating VILI [19–22]. The release by injured cells of mitochondrial alarmins, such as mitochondrial DNA, *N*-formyl-methionyl-leucyl-phenylalanine (fMLP), and adenosine triphosphate (ATP) produces a local and sometimes systemic inflammatory response. This response is mainly dependent on the local production of interleukin-1ß (IL-1ß) *via* the assembly of the NOD-like-receptor protein 3 (NLRP3) inflammasome and the recruitment of neutrophils to injured tissues [16–18, 23]. Since IL-1ß is a prominent and bioactive pro-inflammatory cytokine in the lower airways from patients with ARDS [6, 7, 24], we hypothesized that mitochondrial alarmins may be released by airway cells submitted to cyclic stretch due to mechanical ventilation, and represent the missing link between cell stretch and downstream inflammatory cytokines.

## Methods

### *In vitro* cell stretching

The human alveolar type II-like A549 cells (ATCC, Manassas, VA) were cultured onto silastic membranes (Bioflex® plates, Dunn Labortechnik, Asbach, Germany), and submitted to cyclic stretch using the FX-3000 Flexcell® system (Flexcell International, Hillsborough, NC, USA), as previously described [13, 25]. In some experiments, we substituted A459 cells for primary human monocyte-derived macrophages [8]. In other experiments, macrophages submitted to cyclic stretch were co-incubated with 100 ng/mL of *E.coli* K12 LPS (UltraPure® LPS, Invivogen). ATP (Roche) was used at 100 μM concentration, and the Toll-like receptor 9 (TLR9) antagonist ODN TTAGGG (Invivogen) was used at 1 μM concentration. Detailed methods are described in the online data supplement. Cell viability before and after cell stretching was assessed using phase contrast optic microscopy and fluorescence microscopy (Live/Dead kit®, Thermo Fisher Scientific, USA), and by flow cytometry (FACS, 7-AAD, BioRad). Briefly, cells were incubated for 15 min in the fluorescently-labeled calcein-AM (live cells, green) and propidium iodide (dead cells, red) solution, and analyzed by fluorescent microscopy. FACS analysis of static vs. stretched cells was done using the 7-AAD reagent (BioRad) staining live cells, excluding dead cells.

### Measurement of alarmins in conditioned supernatants

#### Mitochondrial DNA

For mitochondrial DNA isolation, collected conditioned media from A549 cells and BAL were first centrifuged at 2000 x g for 10min to remove cell debris, followed by DNA extraction using Qiagen DNAEasy kit (QIagen, Hilden, Germany). Quantitative PCR was used to measure levels of mitochondrial DNA(mtDNA) in cell-free supernatants and BAL using mitochondrial specific PCR primers for cytochrome B, cytochrome C oxidase subunit III (COXIII), and NADH dehydrogenase subunit I (ND1) as previously described [17]. In addition, we performed droplet digital PCR (ddPCR) that allowed us to determine the copy number of mtDNA and genomic DNA (gDNA) by amplifying mitochondrial COX III and genomic ribonuclease protein subunit p30 (RPP30) respectively.

In some experiments, 2 μM of the TLR9 antagonist ODN TTAGGG (Invivogen, San Diego, CA, USA) was added to the A549 cell cultures just before starting cell stretch. IL-8 secreted in conditioned supernatants was measured as a marker of cell activation.

#### Cell reporter assay

In order to study TLR9 activation, supernatants from stretched cells were added to HEK293 Blue® cells expressing TLR9 (Invivogen). HEK293 Blue® cells respond to TLR9 agonists such as bacterial and mitochondrial DNA by secreting embryonic alkaline phosphatase (SEAP) [26, 27]. Cell activation was measured using an alkaline phosphatase substrate present in the colorimetric HEK-Blue^TM^ detection system (Caya-Invivogen Europe, Toulouse, France), quantified using an ELISA reader at 655 nm, and expressed as optical densities. Those experiments were repeated with human bronchoalveolar lavage (BAL) samples.

#### ATP

Extracellular ATP released by A549 cells was quantified in conditioned supernatants using the ATP bioluminescence assay kit CLS II with a detection range of 10^−11^–10^−6^ M (Roche Applied Science, Mannheim, Germany). For optimal measurements with this method, pH of the samples was set at pH 7.7 using Tris buffer. A standard curve was performed with purified ATP.

#### Chemotactic factors

Chemotaxis of human neutrophils induced by supernatants from stretched A549 cells was measured using a modified Boyden chamber as previously described [28]. For each experiment, serial dilutions of prototypical chemotactic factors, such as human IL-8 (a gift from C. Power, MerckSerono, Geneva, Switzerland) and the bacterial/mitochondrial formylated peptide fMLP (Sigma, St. Louis, MO) served to control a maximal rate of neutrophil chemotaxis. In some experiments, neutrophils were pre-incubated 30 min prior to start the chemotaxis assay with an anti-formyl peptide receptor 1 (FPR1) monoclonal mouse antibody (blocking the human surface receptor for fMLP, R&D, Minneapolis, MN, USA) or an anti-C-X-C motif chemokine receptor 1 (CXCR1) blocking antibody (Abcam). More detailed methods are described in the online data supplement.

#### IL-8 and IL-1ß

Conditioned supernatants collected after A549 cell stretching were centrifuged at 1,200 rpm for 5 min at 4°C to remove cell debris. IL-8 levels were measured in supernatants using a sandwich ELISA using a pair of monoclonal antibodies as previously described [25]. IL-1ß levels were measured in conditioned supernatants and in cell lysates from monocyte-derived macrophages (cell lysed using a Triton-X-based cell lysis buffer) using a sandwich ELISA (Perbio Science Switzerland SA). This antibody pair does not differentiate pro-IL-1ß from mature IL-1ß. Monocyte-derived macrophages were also cultured with the TLR9 agonist (ODN TTAGGG, Invivogen), ATP, or both. The secretion of IL-1ß in conditioned supernatants from monocyte-derived macrophages was also assessed using a Western blot technique as described by Wu et al. [22].

### Mechanical ventilation in rabbits

#### Animals

Male New Zealand white rabbits (3.0 to 3.3 kg) were obtained from the “élevage scientifique des Dombes” (Romans, France) and bred in the University of Burgundy animal facility (Dijon, France). They were placed in individual cages, had free access to water, and were fed in accordance with current recommendations described in the *Guide for the Care and Use of Laboratory Animals*, National Institutes of Health No. 92–23, revised version of 1985. The protocol for animal studies was approved by the local veterinary committee (Ethics committee for animal research, C2EA grand campus of Dijon #105). Experiments were performed according to European laws and regulations on animal welfare. A central venous catheter was surgically inserted into every rabbit the day before MV.

#### Experimental protocol

Twenty-four hours after jugular catheterization, animals were orally intubated with a 2.5mm tracheal cuffed tube (Mallinckrodt™, Covidien®, U.S.A.) under general anesthesia obtained with ketamine 20 mg/Kg (Panpharma, France) and xylazine 1,5 mg/Kg (Rompun®, Bayer, Germany). Animals were put in the supine position on a heating blanket and connected to a volume-controlled ventilator (Servo ventilator 900C, Siemens®, Germany) (20 mL/kg of tidal volume with zero end-expiratory pressure [ZEEP], a respiratory rate of 30 bpm and a fraction of inspired oxygen at 0.5). Ventilated rabbits were perfused with isotonic saline and kept anesthetized and paralyzed throughout the experiment with 0.2mg/kg/h midazolam (Hypnovel®, Roche, Switzerland) and, 0.8mg/kg/h cisatracurium besilate (Nimbex®, GlaxoSmithKline, U.K). Animals were sacrificed after 4 hrs. or 8 hrs. of mechanical ventilation (MV). Intubated, but spontaneously breathing (SB) rabbits were used as controls (n = 8). At the end of the experiments, animals were exsanguinated by venous puncture, and autopsied aseptically to harvest the lungs. Bronchoalveolar lavage (BAL) followed by surgical removal of the lungs was performed at the end of the experiment as described [25]. Briefly, BAL was performed in the lower pulmonary lobe using an appropriate catheter with 5 mL of sterile 0.9% NaCl. The collected BAL fluid was divided into aliquots, and stored at -80°C until analyses were performed. The remaining tissue was homogenized in sterile water. Lung homogenates were then frozen, and stored at -80°C until tissue concentrations of cytokines were measured. Measurement of inflammatory mediators in BAL fluids and in lung tissues, as well as the assessment of a histological lung injury score was done as described elsewhere [25]. More detailed methods can be found in the online data supplement, including the quantitative measurement of mitochondrial DNA in BAL fluids. Methods for the quantification of chemotactic attraction of human neutrophils by rabbit BAL fluids are also described in the online data supplement.

### Patients with ARDS

BAL fluid was obtained through fiberoptic bronchoscopy in intubated and mechanically ventilated patients at day 1 and day 7 after the onset of ARDS (Harborview Medical Center, Seattle, WA, USA) [29] after having obtained an informed consent from the patient or the next-of-kin. BAL fluid was also obtained in 3 healthy volunteers, centrifuged and kept frozen at -80°C [29]. Total DNA was extracted from cell-free human BAL fluids and quantitative PCR was done measuring levels of the mitochondrial cytochromes B and C oxidase III DNA, as described above for supernatants from stretched cells. BAL samples were defined as “highly inflammatory” if they contained > 80% neutrophils and > 2 mg/mL proteins. Others were defined as “low inflammatory”.

### Statistical analysis

Data are presented as median with interquartile range. Because the majority of the data were not normally distributed or had low sample size, non-parametric tests were used. The Mann-Whitney *U* test or the Kruskall-Wallis test were used to compare variables between two or multiple groups respectively. To account for multiple comparisons, the *P*-value was adjusted for a false discovery rate (FDR) using the Benjamini and Hochberg method. A FDR (or q-value) of 0.05 was considered significant. Data were analyzed with the GraphPad Prism® software [30].

## Results

### Human type II-like alveolar epithelial cells submitted *in vitro* to cyclic stretch release mitochondrial DAMPs

**Fig 1:**
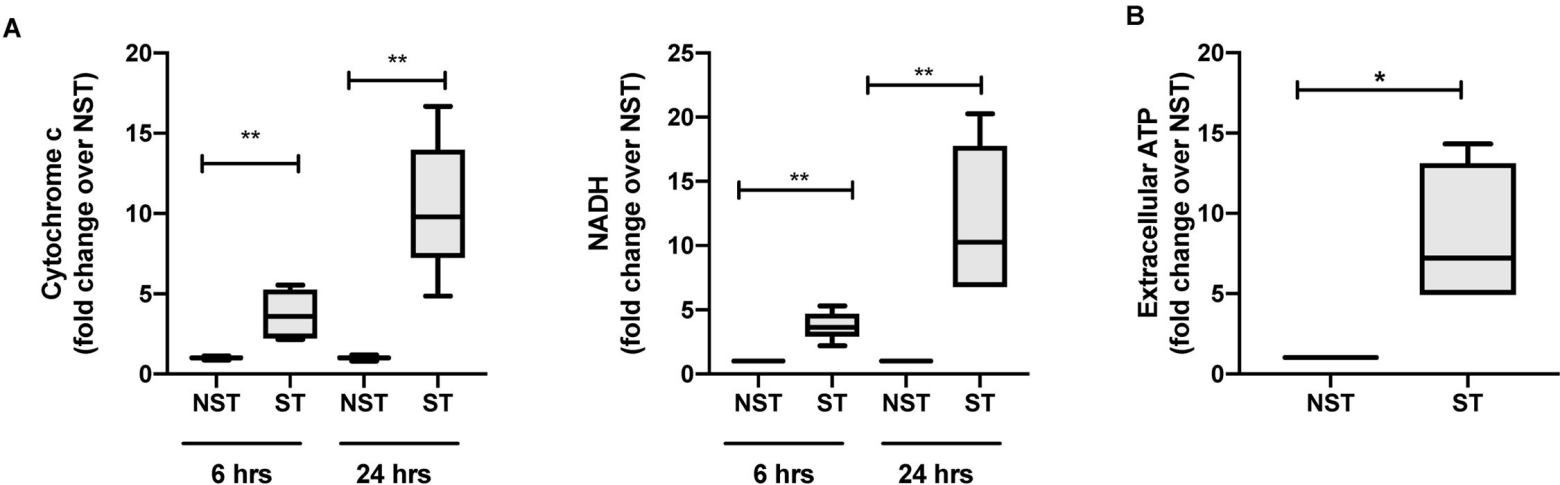
Mitochondrial DAMPs are released during cyclic mechanical stretch. (**A**)Mitochondrial DNA (cytochrome C oxidase III and NADH) release in conditioned supernatants from human alveolar type II-like epithelial A459 cells submitted to cell stretching for 6 and 24 hours or kept in static conditions, expressed as fold changes over non stretched cells.(**B**) Extracellular ATP quantified by luciferase assay in conditioned supernatants from human alveolar type II-like epithelial A459 cells submitted to cell stretching for 24 hours, and from cells kept in static conditions, expressed as fold changes over non stretched cells. Data represent median with interquartile range of at least three independent experiments. *P < 0.05, **P < 0.01, Mann-Whitney test (**A-B**). NST: non stretch, ST: stretch, NADH: nicotinamide adenine dinucleotide.

First, we investigated whether mitochondrial DAMPs are released during mechanical stretch using an *in vitro* model of mechanical ventilation. We found that mitochondrial DNA levels were significantly increased in culture supernatant from A549 cells exposed to mechanical stretch as compared to unstretched cells for all stretch periods (Fig 1A). This result was further validated by digital droplet PCR showing a significant increase in the mtDNA/gDNA ratio in the supernatant of cells exposed to 24h stretch as compared to unstretched cells (S1 Fig). In addition to mtDNA, we also measured an increase in extracellular ATP in supernatants from A549 cells exposed to 24 hrs. of stretch as compared to unstretched cells (Fig 1B). Although little differences were observed in stretched cells using phase contrast and fluorescent microscopy, we measured by FACS a small increase in the number of dead cells (or cells with permeable/ruptured membranes) after cell stretching compared with cells kept in static conditions (S2 Fig). These data demonstrate that mitochondrial DAMPs are released in the extracellular space during cyclic mechanical stretch.

### Conditioned media from cell exposed to stretch activates TLR9 signaling

**Fig 2:**
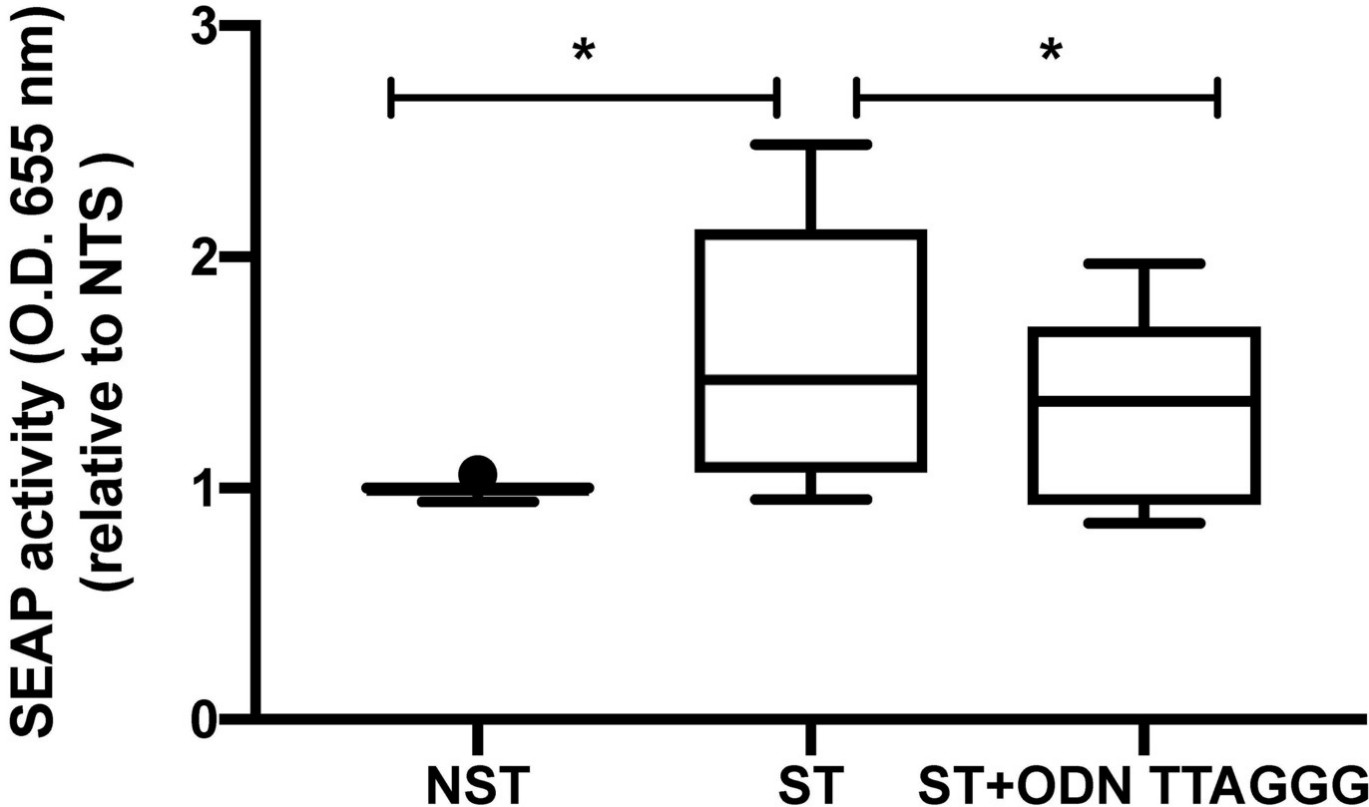
Supernatant from 24 hours stretched A549 cells induces TLR9 signaling that can be blocked with TLR9 antagonists. Supernatant collected from 24 hours stretched A549 cells and from unstretched cells were co-incubated with HEK-Blue hTLR9 reported cell line either alone or with TLR9 antagonists ODN TTAGGG. After 24 hours supernatants were analyzed for activity by spectroscopy of the target transgene NF-κB induced secreted embryonic alkaline phosphatase absorbance at 655 nm. Data are expressed as fold change over NST and represent median with interquartile range of at least three independent experiments. *P < 0.05, Mann-Whitney test (NST vs ST) and Wilcoxon signed-rank test (ST vs ST+ ODN TTAGGG) with FDR correction. NST: non stretch, ST: stretch.

To determine whether the mitochondrial DAMPs released by mechanical stretch are biologically active, we exposed HEK Blue^TM^ hTLR9 reporter cell line to the supernatant from stretched and unstretched cells. Supernatant from stretched cells induced a significant increase in TLR9 signaling as measured by SEAP production as compared to supernatant from unstretched cells (Fig 2). Co-incubation of conditioned media from stretch cells with TLR9 antagonist ODN TTAGGG caused a decrease in SEAP production. These data demonstrate the presence of biologically active TLR9 agonists in conditioned media from cells exposed to mechanical stretch.

### Mechanical stretch causes the extracellular release of IL-8 *via* a TLR9 dependent mechanism

**Fig 3:**
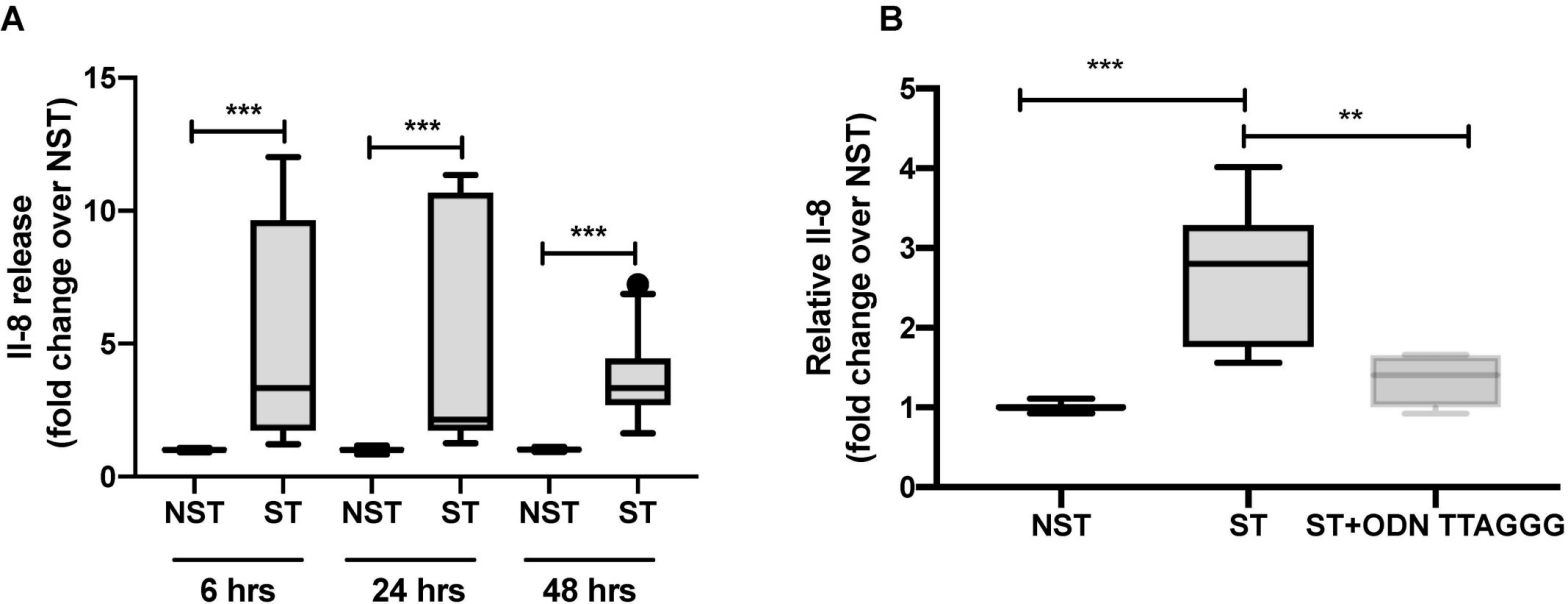
Mechanical stretch induces extracellular secretion of pro-inflammatory cytokine Il-8 that can be blocked with TLR9 antagonists. (**A**) Il-8 concentration measured in supernatant collected from A549 cells submitted to cyclic mechanical stretch for 6, 24 and 48 hours and their control unstretched cells. (**B**) Il-8 concentration measured in supernatant collected from A549 cells stretched for 24 hours and co-incubated with TLR9 antagonist ODN TTAGGG. Data are expressed as fold change over NST and represent median with interquartile range of at least three independent experiments. **P < 0.01, ***P < 0.001, Mann-Whitney test (**A**), Kruskall-Wallis test with FDR correction (**B**). NST: non stretch, ST: stretch.

Next, we explored whether mtDAMPs may be involved in stretch-induced IL-8 secretion through TLR9 signaling. We measured the production of the IL-8 chemokine in media from cells exposed to stretch and co-incubated with the TLR9 antagonist ODN TTAGGG. We found that IL-8 concentrations increased in the supernatant from stretched cells as compared to unstretched cells for every time period in a time-dependent manner (Fig 3A). Interestingly, stretch-induced IL-8 secretion decreased with co-incubation with a TLR9 antagonist (Fig 3B).

Since mtDNA promotes TLR9-mediated inflammation, these data suggest that mtDNA may represent a potential TLR9 agonist in our *in vitro* stretch model. Once released in the extracellular milieu by stretch, mtDNA may elicit a paracrine pro-inflammatory effect through TLR9 signaling.

### Mechanical stretch induces the production and secretion of IL-1β by stretched monocyte-derived macrophages

**Fig 4:**
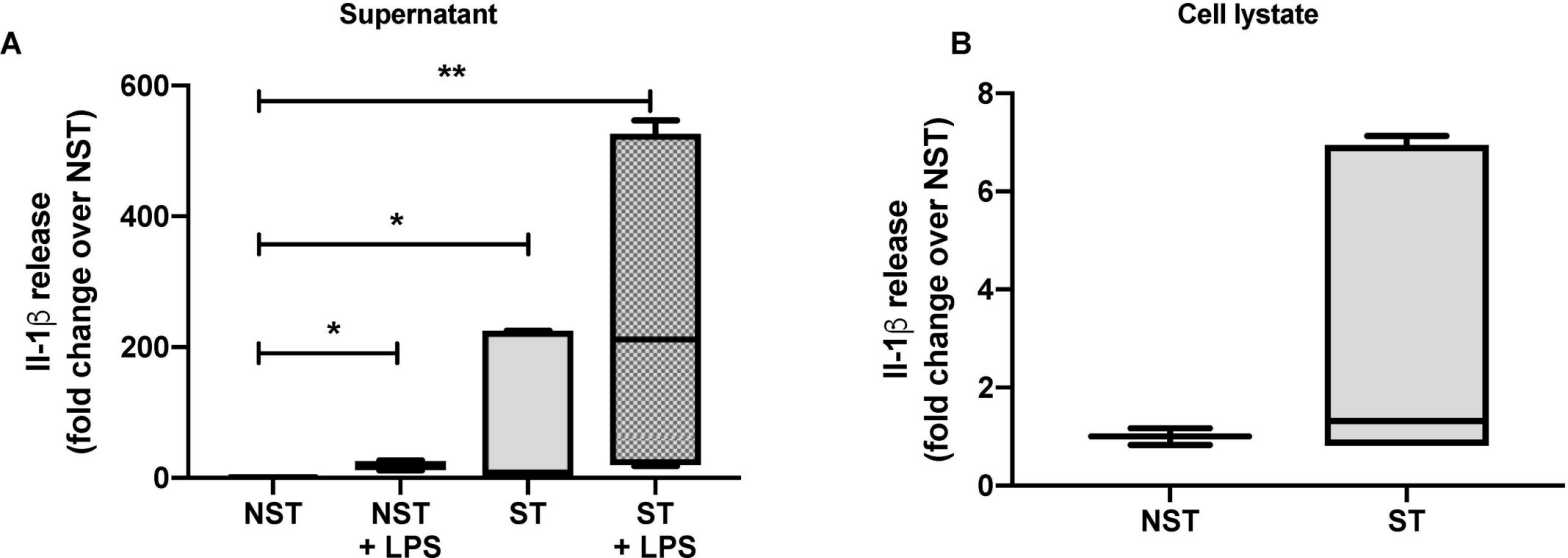
Mechanical stretch and TLR4 agonists LPS have a synergistic effect on pro-inflammatory cytokines Il-1β secretion in monocytes derived macrophages submitted to mechanical stretch. (**A**) Il-1β concentration measured by ELISA in supernatant isolated from monocytes derived macrophages submitted to 24 hours cyclic mechanical stretch and/or co-incubated with LPS. (**B**) Il-1β concentration measured by ELISA in cell lysates from monocytes derived macrophages submitted to 24 hours mechanical stretch and unstretched cells. Data are expressed as fold change over NST and median with interquartile range of at least three independent experiments. *P < 0.05, **P < 0.01, Kruskall-Wallis test with FDR correction (**A**), Mann-Whitney test (**B**). NST: non stretch, ST: stretch, LPS: lipopolysaccharide.

Lung macrophages are tissue-resident immune cells that play a critical role in maintaining homeostasis and fighting infection in the lungs. We investigated whether mechanical stretch could synergistically activate macrophages with TLR agonists either directly and/or indirectly. IL-1ß concentrations were measured in conditioned media, and in cell lysates from activated macrophages. Using immunoblotting and ELISA, we found that IL-1ß concentration significantly increase in supernatant from monocyte-derived macrophages submitted to 24 hrs. of mechanical stretch as compared to unstretched cells (Fig 4A). However, there was no difference in IL-1β production in cell lysates of stretched monocyte-derived macrophages as compared to unstretched monocyte-derived macrophages (Fig 4B). Interestingly, we found a synergistic effect between mechanical stretch and the TLR4 agonist LPS for IL-1ß production and secretion in supernatant and cell lysates from monocyte-derived macrophages. These experiments suggest that mechanical stretch induces the secretion of IL-1ß by monocyte-derived macrophage with a synergistic effect with LPS.

### Conditioned media from stretched A549 cells induces chemotaxis of human neutrophils

**Fig 5:**
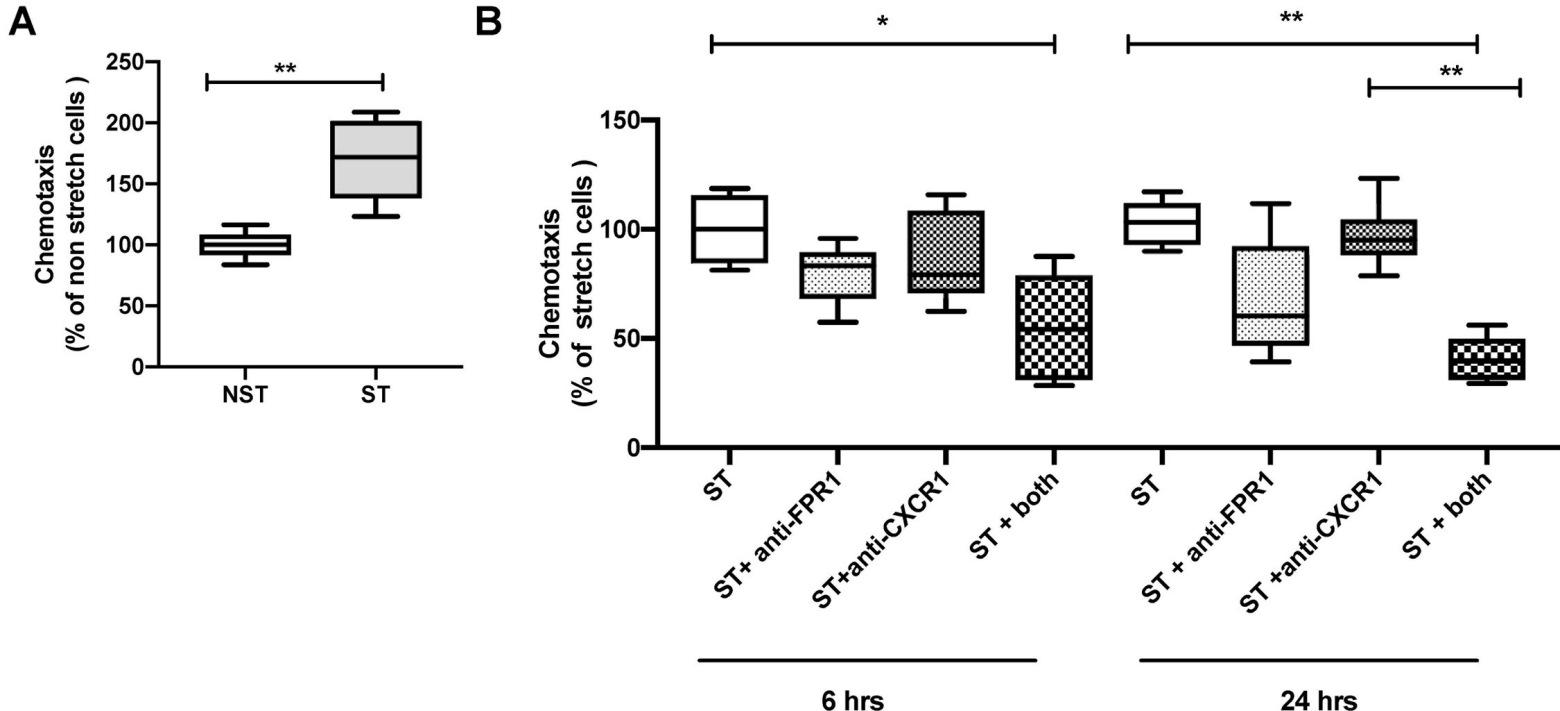
Supernatant from stretched A549 cells induces neutrophils chemotaxis mediated principally by chemoattractant fMLP. Boyden chamber chemotaxis assay of human primary neutrophils stimulated with (**A**) conditioned supernatants collected from A549 cells submitted mechanical stretch for 24 hours and unstretched A549 cells and (**B**) antibodies against the fMLP receptor (anti-FPR1) and the IL-8 receptor (anti-CXCR1). Data are expressed as percentage of NST (**A**) and ST (**B**) and represent median with interquartile range of at least three independent experiments. *P < 0.05, **P < 0.01, Mann-Whitney test (**A**), Kruskall-Wallis test with FDR correction (**B**). NST: non stretch, ST: stretch, FPR1: anti-formyl peptide receptor 1, CXCR1: C-X-C motif chemokine receptor 1.

Since IL-8 and fMLP are both neutrophil chemokines, we evaluated next the chemoattractant properties of supernatants collected from A549 cells exposed to cyclic mechanical stretch. Conditioned media from A459 cells exposed to mechanical stretch for 24 hrs. induced chemotaxis of human neutrophils as compared to unstretched cells (Fig 5A). Blocking formyl peptide receptors or CXR1 receptors alone were not sufficient to decrease stretch-induced chemotaxis (Fig 5B). Interestingly when both receptors were blocked, we measured a significant decrease in chemotaxis after both 6 and 24 hrs of mechanical stretch. Those results suggest that mitochondrial fMLP may be released by A549 cells during stretch, and may act synergistically with Il-8 to induce neutrophil chemotaxis.

### Lung inflammation and alarmins in rabbits submitted to adverse mechanical ventilation

**Fig 6:**
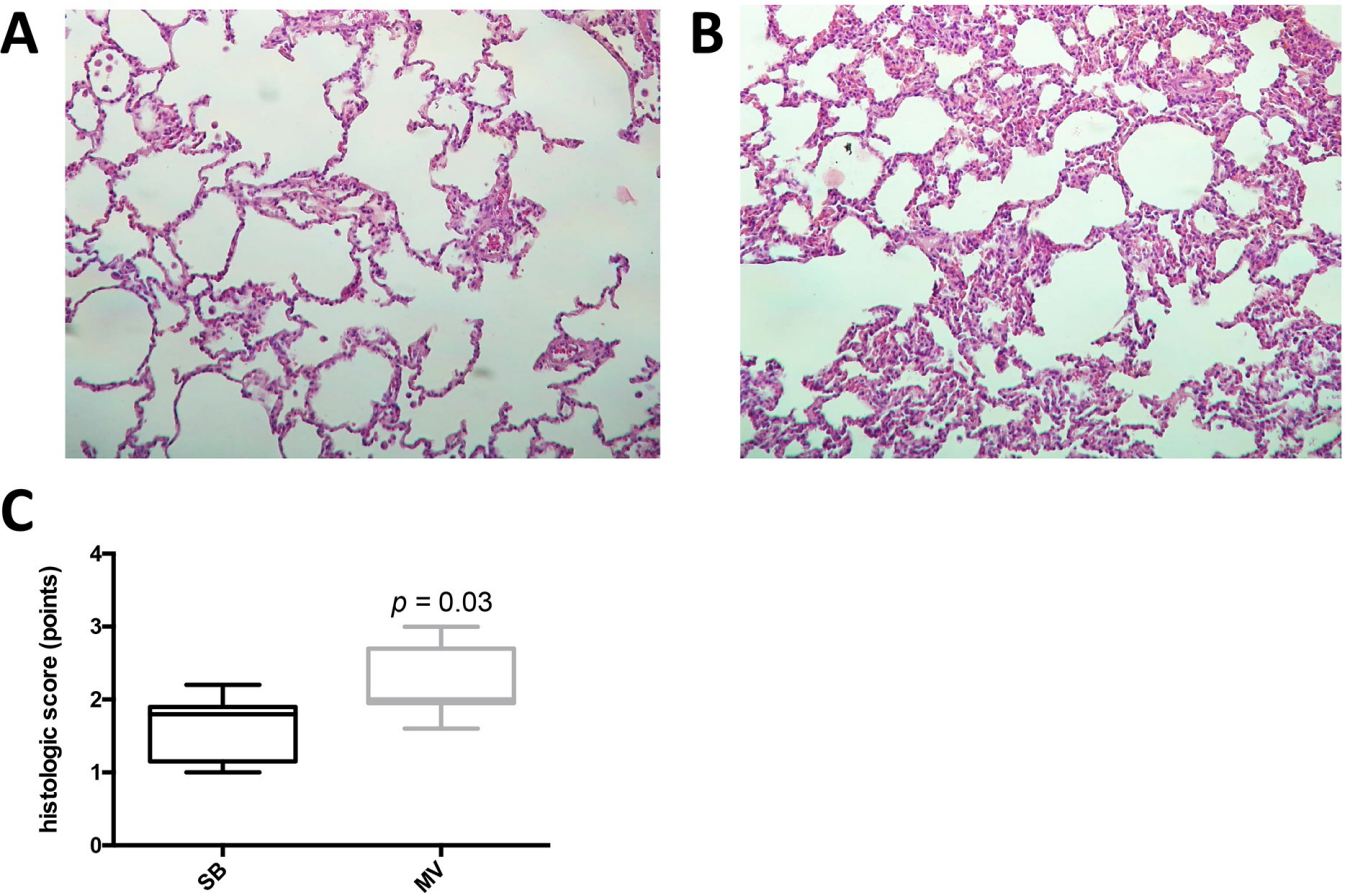
Rabbits exposed to injurious mechanical ventilation present signs of lung injury characterized by massive neutrophil infiltration. Representative lung histology (hematoxylin-eosin stain) in spontaneously breathing rabbits (**A**) and in rabbits submitted to mechanical ventilation for 4 hours (**B**). Histological lung injury score in rabbits submitted to mechanical ventilation compared to spontaneously breathing (SB) animals. Tissue injury was scored based on the degree of polymorphonuclear infiltration, hemorrhage, and edema in the interstitial and alveolar spaces on a scale from 0 to 3 points (**C**). Data represent median with interquartile range of at least three independent experiments. *P < 0.05* as indicated, Mann-Whitney test. MV: mechanical ventilation, SB: spontaneously breathing.

**Fig 7:**
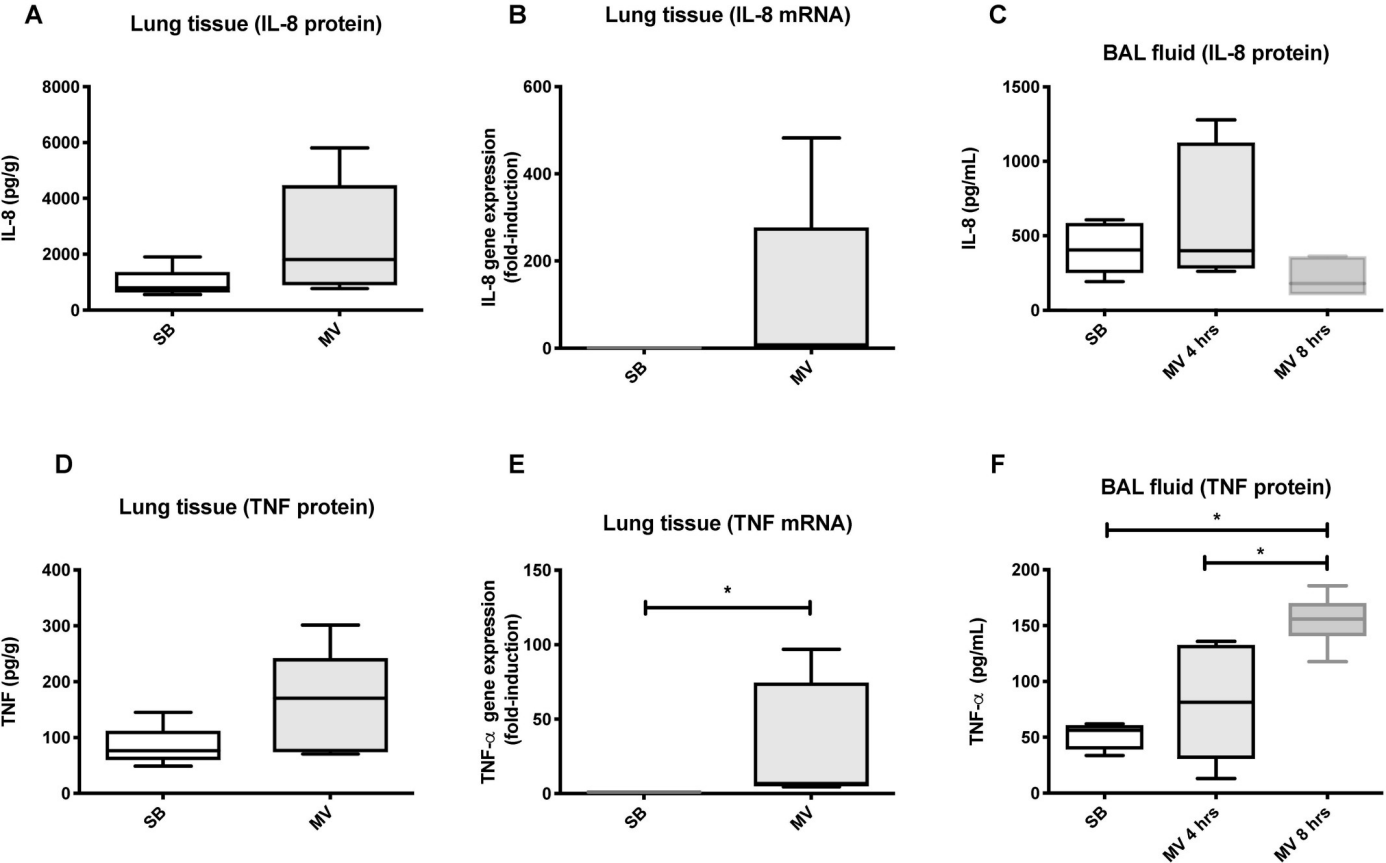
Injurious mechanical ventilation induces a pro-inflammatory response in the lungs of ventilated rabbits. Lung production (lung tissue mRNAs and proteins) and secretion into the alveolar space (bronchoalveolar lavage [BAL] fluid) of the IL-8 chemokine (**A**, **B**, and **C**) and the pro-inflammatory cytokines TNF-α (**D**, **E**, and **F**) by rabbits submitted to mechanical ventilation and by spontaneously breathing animals. Data represent median with interquartile range of at least three independent experiments. *P < 0.05, Mann-Whitney test (**A,B,D,** and **E**), Kruskall-Wallis test with FDR correction (**C** and **F**). MV: mechanical ventilation, SB: spontaneously breathing.

**Fig 8:**
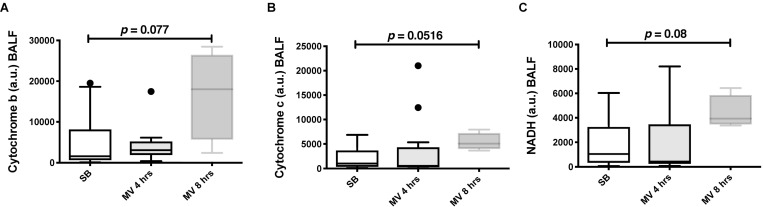
Injurious mechanical ventilation causes the extracellular release of mitochondrial DNA in ventilated rabbits. Mitochondrial DNA (cytochrome B, [**A]**; cytochrome C oxidase III, [**B]** and NADH, [**C]**) measured by quantitative PCR in bronchoalveolar lavage (BAL) fluid from rabbits submitted to mechanical ventilation for 4 and 8 hours and in spontaneously breathing animals. Data represent median with interquartile range of at least three independent experiments. Kruskall-Wallis test with FDR correction (**A-C**). MV: mechanical ventilation, SB: spontaneously breathing.

We next tested whether our *in vitro* findings could be translated into a rabbit model of VILI. In the rabbit model, an injurious mechanical ventilation (MV) regimen induced significant lung injury and neutrophil recruitment, as compared with control spontaneously breathing (SB) animals (Fig 6A, 6B and 6C). Despite the development of lung injury, pro-inflammatory cytokines were only mildly elevated in the lung tissue of ventilated rabbit as compared to SB animals. MV increased TNF-α gene expression and BAL concentration, but not Il-8 (Fig 7). Mitochondrial DNA could be detected in BAL fluids from both SB and MV animals (Fig 8A, 8B and 8C). Although mitochondrial DNA concentration increased overtime in BAL from MV animals as compared to SB animals, the difference was not statistically different.

**Fig 9:**
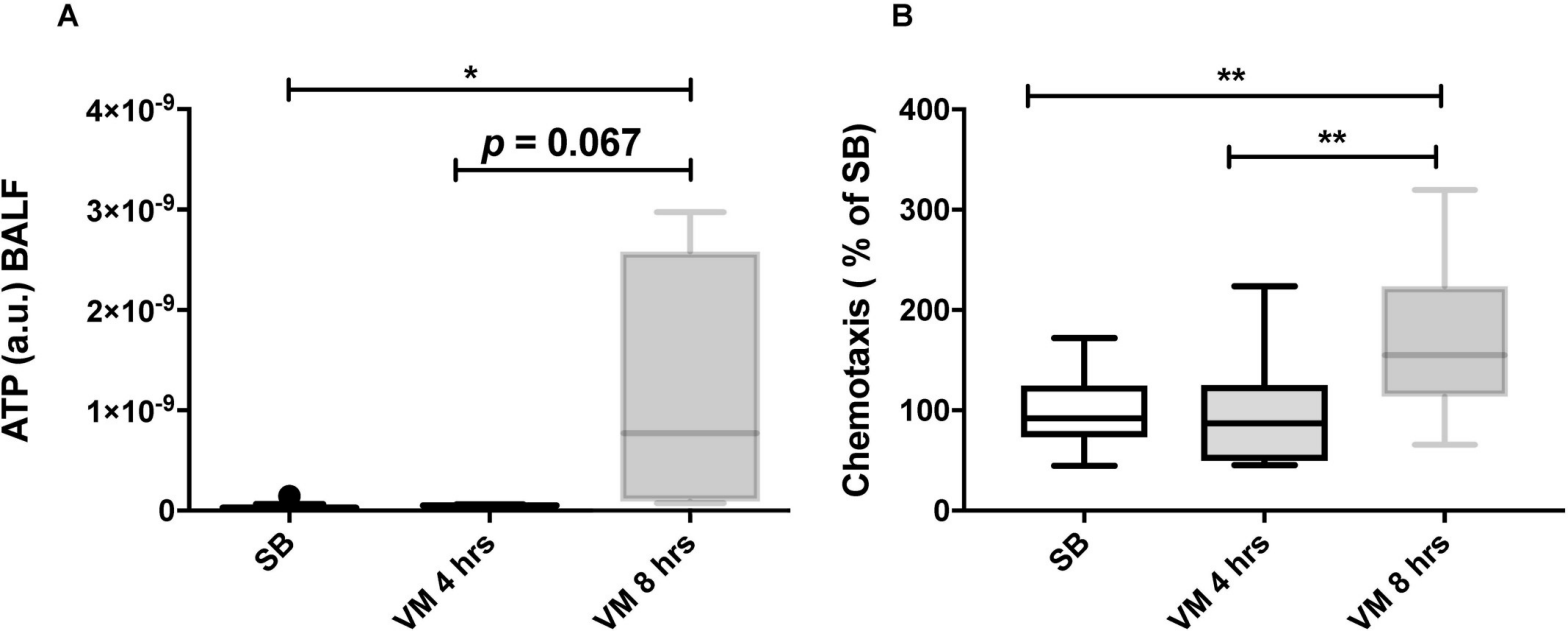
BAL fluid from ventilated rabbits presents elevated ATP level and has chemotactic activity. (**A**) Extracellular ATP measured in bronchoalveolar lavage (BAL) fluid from rabbits submitted to mechanical ventilation for 4 and 8 hours and in spontaneously breathing animals. (**B**) Chemotactic activity of BAL fluid from rabbits submitted to mechanical ventilation for 4 and 8 hours and in spontaneously breathing animals. Human primary neutrophil chemotaxis was measured in a 96-well Boyden chamber and expressed as percentage migration compared to SB. Data represent median with interquartile range of at least three independent experiments. *P < 0.05, **P < 0.01, Kruskall-Wallis test with FDR correction (**A-B**). MV: mechanical ventilation, SB: spontaneously breathing.

Significantly higher ATP was found in BAL fluids from rabbits subjected mechanically ventilated for 8 hrs. (Fig 9A). Similar to supernatants from stretched A549 cells, BAL fluids from MV animals induced *in vitro* chemotaxis of human neutrophils, particularly in BAL fluids originating from animals ventilated for 8 hrs. (Fig 9B). Taken together, these results suggest that mitochondrial alarmins are released in the lower airways of rabbits submitted to an injurious MV and induces neutrophils chemotaxis. Further studies will be required to determine whether the release of mitochondrial DAMPs during mechanical ventilation is caused by an active process or the result of cell death.

### Mitochondrial DNA in bronchoalveolar lavage fluids from ARDS patients

Finally, we investigated the human relevance of our experimental and animal data by analyzing BAL fluids from human patients with ARDS.

**Fig 10:**
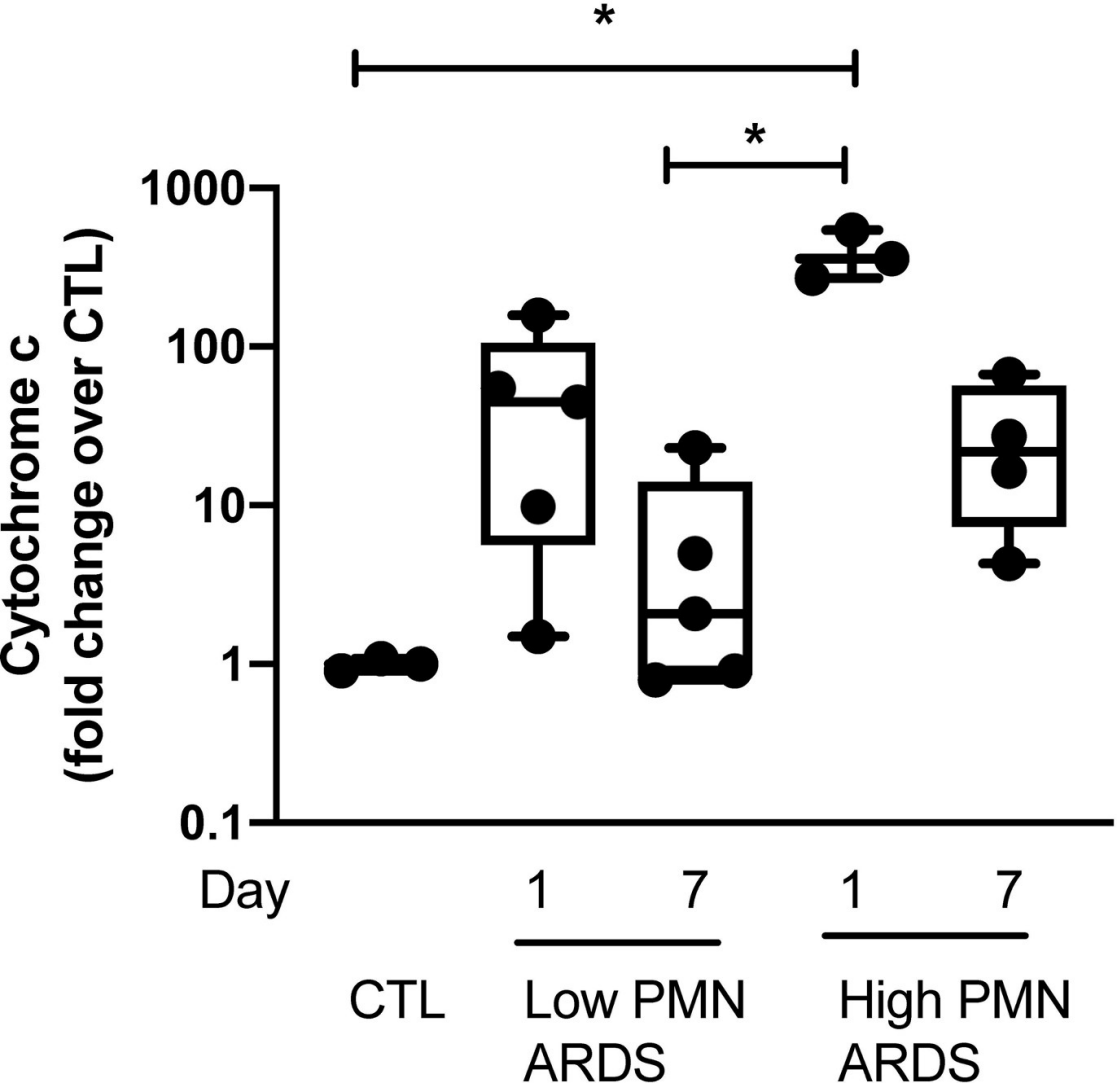
Mitochondrial DNA are released in BALF of patients with ARDS. Mitochondrial DNA (cytochrome C oxidase III) measured by quantitative PCR in bronchoalveolar lavage fluid of patients with ARDS at day 1 and day 7 of presentation and healthy volunteers. Patients with ARDS were categorized as “highly inflammatory” (high PMN) if they contained > 80% neutrophils and > 2 mg/mL proteins or “low inflammatory” (low PMN) if they did not meet those criteria. Data are expressed as fold change over healthy volunteers and represent median with interquartile range of at least three independent experiments. *P < 0.05, Kruskall-Wallis test with FDR correction (**A-B**). CTL: controls (healthy volunteers), PMN: polymorphonuclear, ARDS: acute respiratory distress syndrome.

Mitochondrial DNA was detectable by qRT PCR in BALF from patients with low and high inflammatory ARDS, defined as a neutrophil count of > 80% and protein levels > 2 mg/mL. Mitochondrial DNA concentration was the highest in the BALF from patients on day 1 of high inflammatory ARDS as compared to healthy volunteers and declined at day 7 of ARDS (Fig 10).

BAL fluids from ARDS patients at Day 1 with a high inflammatory profile induced TLR9 signaling in exposed HEK Blue™ hTLR9 reporter cells (S3A Fig), and this activity was inhibited by the TLR9 antagonist ODN TTAGGG (S3B Fig).

## Discussion

Herein, we show that mitochondrial DNA, ATP and fMLP are released from human type II-like A459 lung alveolar cells submitted to cyclic stretch. Similarly, the same pro-inflammatory alarmins are found in the airways of rabbits submitted to a short injurious MV regimen, and mitochondrial DNA concentrations were found to be markedly elevated in BAL fluids from patients with ARDS. Conditioned media from stretched cells and BAL from ventilated rabbits were shown to induce neutrophil chemotaxis, attributable to the mitochondrial fMLP chemokine with a possible synergistic effect with Il-8. In addition, conditioned media from stretched cells could modulated stretch-induce IL-8 secretion through TLR9 signaling, suggesting that mtDNA may be a TLR9 agonist in this system.

Lung inflammation is a hallmark of acute lung injury and ARDS [7, 31]. Lung inflammation originates from the primary lung insult (pneumonia, sepsis, gastric fluid aspiration, trauma, etc.). It has become evident that MV could *per se* induce airway inflammation, and more importantly synergized with the primary lung injury. Supporting this, ARDS patients receiving lung protective ventilator strategy have lower levels of alveolar and systemic inflammation, in particular less alveolar neutrophils, and lower levels of the pro-inflammatory cytokines IL-1ß and TNF-α [32].

Recent studies demonstrated that during cell death and organ injuries, certain molecules released from mitochondria such as mtDNA, fMLP and ATP may function as DAMPs and activate the innate immune system [17, 33]. The role of mitochondrial DAMPs in the pathogenesis of lung inflammation were highlighted in an animal study by Zhang et al [34], who demonstrated that intravenous mtDNA injection in rats induced acute lung injury through activation of the TLR9/NF-κB signaling pathway.

In the present study, we demonstrated that mechanical stretch causes extracellular release of mitochondrial DNA and ATP in both our experimental and animal model of MV. Cellular necrosis and disruption of cytoplasmic membrane may constitute a potential mechanism for the extracellular release of mitochondrial DAMPs in our stretch model. However, recent experimental and human studies with trauma patients [35, 36] have demonstrated that extracellular release of mitochondrial DNA in absence of markers of cellular necrosis, suggesting another mechanism for mitochondrial DAMPs release after trauma. Experimental studies of mitochondrial injury have shown that disruption of mitochondrial membrane integrity lead to leakage of free-mtDNA fragments into the cytosol followed by extracellular release [37]. In addition, mitochondrial components of injured mitochondria may also be actively released into the extracellular milieu through autophagy [38] and mitochondrial derived vesicles [39], without disruption of the cytoplasmic membrane integrity. Since mitochondria anchor to the cytoskeleton, they may also function as mechanotransducers and respond directly to cyclic mechanical stretch. This was suggested by Ali et al. [40] who showed that mechanical strain in endothelial cells induces reactive oxygen species (ROS) production by mitochondria that was abrogated after treatment with cytochalasin D, a known disruptor of cytoskeletal network. In our in vitro experiments, no significant necrosis or apoptosis was observed in stretched cells. This suggests that stretch-induced mtDAMPs release in our study may be the result of an active process triggered by cyclic strain rather than the result of disruption of cellular integrity.

Plasma release of cell-free mtDNA has been shown to occur in patients with trauma and sepsis [18]. In VILI animal models mtDNA release has been inconstantly found. Increased mtDNA has been measured in BAL fluids from mice in an acid-induced lung injury model [41], as well as in rats exposed to injurious ventilation [42]. In other MV-induced lung injury in mice [43] and rabbits [44], MV was not associated with an increase in mtDNA concentrations in BAL fluid. In our model, mtDNA was released within the airway from ventilated rabbits but there was high variability in mtDNA content among different animal groups for the three mitochondrial primers tested. A possible explanation for these apparent discrepant results is that levels of lung injury were different in these VILI models, with lower tidal volumes [44], and protective PEEP [43]. Difference in mtDNA concentration in BAL fluid among different VILI models may also be explained by heterogeneity in cellular capacity to restore mitochondrial density following injury resulting in progressive loss of intracellular mtDNA copy number. This was demonstrated by Haden et al.[45] in a murine model peritonitis induced by *Staphylococcus aureus*, showing that initial mitochondrial injury and loss of mitochondrial DNA copy number could be restored through the activation of mitochondrial biogenesis.

After demonstrating that mechanical stretch causes extracellular release of mtDNA and ATP, we explored next whether mitochondrial DAMPs released by stretch were biologically active. Mitochondrial DNA activates the immune response directly *via* the activation of TLR9 pathways resulting in a pro-inflammatory response [37]. Zhang et al. showed that mitochondrial DNA injected IV to rats induced lung inflammation, increased permeability of the alveolar capillary barrier [17], and upregulated TLR9 in lung tissue [34]. In line with those findings, we show that conditioned media from stretched A549 cells as well as BAL fluid from ventilated rabbits both triggered TLR9 signaling in a HEK-Blue hTLR9 reported cell line. Interestingly, mitochondrial DNA seems to be more potent as a TLR9 agonist when it is oxidized [46]. The production of reactive oxygen species by different alveolar cells is thought to be an important pathogenic and pro-inflammatory mechanism during ARDS, due to inflammation and hyperoxic environment [47]. Aside from protein and lipid oxidation, oxidation of mitochondrial DNA could then well represent an important pro-inflammatory mediator of acute lung injury. To further support the role of TLR9 signaling in pro-inflammatory response induced by stretch, blockade of the mitochondrial DNA receptor TLR9 by the TLR9 antagonist ODN TTAGGG abrogated the stretch-induced IL-8 secretion by these cells. This strongly suggests autocrine and paracrine cell activation by cytoplasmic alarmins released during cell cyclic stretch.

Polymorphonuclear neutrophil recruitment to the lung and neutrophil-dependent tissue damage have been proposed as key pathogenic factors in VILI and ARDS [5, 48]. It was long thought that the main neutrophil chemotactic and activation factor was the CXC chemokine IL-8. It was however recently shown in a murine model of aseptic thermal hepatic injury that mitochondrial fMLP released by injured cells was the essential chemotactic factor bringing neutrophils to the necrotic lesion, overriding the effect of CXC chemokines [16]. The formylated peptide fMLP found in both bacterial and mitochondrial walls attracts and activates neutrophils either directly by binding to fMLP receptor-1 (FPR1) or indirectly by inducing IL-8 secretion [49]. Chemoattractant properties on human neutrophils could be demonstrated in both conditioned media from stretched cells and in BAL fluid from ventilated rabbits, supporting previous results in rabbit VILI models [44]. Interestingly, the neutrophil chemotactic activity present in supernatants from stretched lung cells could be reduced with FPR1 blocking alone and completely inhibited by combined CXCR1 and FPR1 blockade. This suggests that the main chemotactic factor present in conditioned supernatants from stretched cells was the mitochondrial fMLP.

IL-1ß has been shown to play a major role as a local pro-inflammatory mediator in the context of ARDS and VILI, and is secreted by alveolar macrophages and by alveolar epithelial cells [6, 7, 31, 50]. IL-1ß can be secreted in response to various stimuli, including bacterial molecules such as Gram-negative lipopolysaccharide (LPS). The production of bioactive IL-1ß is tightly regulated and follows a multistep pathway. It requires both the upregulation of the pro-IL-1ß gene expression, and the posttranslational cleavage of pro-IL-1ß into mature IL-1ß. This latter step is dependent on the activation of caspase-1, part of the NALP3 inflammasome complex [51]. It was recently recognized that endogenous danger signals (alarmins) originating from injured/necrotic cells could trigger the production of IL-1ß, and be responsible for aseptic inflammation. Mitochondrial DNA, when released from injured cells, upregulates the IL-1ß gene through its interaction with its receptor, TLR9. Extracellular ATP, which is also released by injured/necrotic cells, induces the assembly of the NALP3 inflammasome and caspase-1 activation through its interaction with the P2X_7_ receptor, resulting in the cleavage of pro-IL-1ß into bioactive IL-1ß [16, 17]. Our results confirm that cyclic stretch of human monocyte-derived macrophages induced the production and the release of IL-1ß, and that cyclic stretch synergized with co-treatment of macrophages with LPS. We therefore postulate that cyclic stretch induces the release by lung cells of mitochondrial DNA (TLR9 agonist), and ATP, and that this leads to the production of IL-1ß by macrophages, and plays an important local pro-inflammatory signal in the lung submitted to (injurious) mechanical ventilation.

Recent work supports the role of NLRP3 inflammasome in the pathogenesis of VILI. NLRP3 inflammasome gene expression was upregulated in ventilated patients, and also in mice ventilated with high tidal volumes, with increased caspase-1 activation, and increased uric acid levels, another NLRP3 agonist [52]. Finally, NLRP3-deficent mice showed less signs of VILI, and IL-1 receptor antagonist partially protected mice against VILI, supporting previously published data [24, 52]. ATP released into the airways during VILI [30] may also therefore be an agonist for NLRP3 in this context. Interestingly, Wu et al. recently reported that mechanical ventilation in mice and *in vitro* alveolar macrophage cyclic stretch induced the assembly of NLRP3 and activated caspase-1, leading to the production and secretion of bioactive IL-1ß [22]. Caspase-1 activation was dependent on the generation of reactive oxygen species by mitochondria, but the possible implication of extracellular ATP as a trigger for inflammasome activation was not directly tested.

Recent clinical studies in patients with sepsis or ARDS suggest that mtDNA levels may be used as a biomarker reflecting the severity of critical illness and predicting mortality. In a large clinical study, Nakahira et al [53], demonstrated that elevated cell-free mtDNA level was associated with ICU mortality. In the present study, we showed that mtDNA could be measured in BAL fluid from patients with ARDS and increased with the degree of lung inflammation. Further studies are needed to address the relevance of alveolar release of mtDNA in ARDS, as a marker or a potential mediator of lung injury.

Our study has some limitations. First, we cannot exclude the participation of bacterial DNA in BAL fluids from patients for TLR9-dependent activation of target inflammatory cells. However, we excluded the presence of bacterial DNA using 16S PCR primers in cell supernatants and in BAL fluids from ventilated rabbits, suggesting that TLR9 was activated in those models by mtDNA. Second, we compared in our animal model an injurious ventilatory regimen (large tidal volume and zero end-expiratory pressure) to spontaneously breathing animals. This choice was made to compare extreme experimental conditions regarding lung cyclic stretch but may be viewed as not clinically relevant. However, in certain circumstances, such as in the operating room, some patients may be ventilated with similar regimens. Future animal studies including non-injurious mechanical ventilation groups will be required to further validate the clinical relevance of our findings. In addition, because of the pulmonary heterogeneity in ARDS, significant portions of the lungs may receive large volumes, despite “protective ventilation” protocols.

In conclusion, our data support a model of VILI based on an aseptic local inflammation due to pro-inflammatory endogenous alarmins released from lung cells injured by airway overstretching, mainly of mitochondrial origin. The presences of mitochondrial alarmins in conditioned media from stretched alveolar epithelial cells and BAL from ventilated rabbits may participate in the pro-inflammatory response induced by mechanical ventilation by attracting neutrophils (fMLP) and stimulating the secretion of pro-inflammatory cytokines by alveolar cells in an autocrine and paracrine manner (mtDNA). These lung alarmins may represent the missing molecular links between the mechanical stress imposed to lung cells, and the observed airway inflammation in critically ill patients submitted to mechanical ventilation. This model also fits with the “danger theory” in which inflammatory and immune cells will respond to mediators of cell injury, rather than to innocuous non-self molecules [54, 55]. It remains to be demonstrated if alarmins released locally by airway stretch act synergistically with other types of lung injury or infection to generate lung inflammation, as suggested by animal studies [11, 25, 56]. It also remains to be tested whether a pharmacologic modulation of alarmin pathways will prevent VILI, and decrease lung inflammation and impairment of pulmonary functions in patients with acute lung injury and ARDS.

## Supporting information

S1 Fig Mitochondrial DNA/genomic DNA ratio increases in supernatant from stretched A549 cells. Mitochondrial DNA (cytochrome C oxidase III) and genomic DNA (RPP30, single copy nuclear gene) measured in conditioned supernatants from human alveolar type II-like epithelial A459 cells submitted to cell stretching for 24 hours or kept in static conditions by digital droplet PCR. Results are expressed as mtDNA/gDNA ratio and represent median with interquartile range of at least three independent experiments. *P < 0.05, Mann-Whitney test. NST: non stretch, ST: stretch, mtDNA: mitochondrial DNA, gDNA: genomic DNA.
(EPS)

S2 Fig Cells exposed to mechanical stretch present not significant morphological difference on phase contrast microscopy examination but a small increase in cellular permeability. A549 cells viability after 24 hrs of cell stretching. Upper panels, phase contrast microscopy; middle panels, fluorescence microscopy (calcein(+)live cells in green, and propidium iodide(+)nuclei of dead cells in red); lower panels, flow cytometry analysis of stretched vs. static cells; 7-AAD(+)dead cells in red, 7-AAD(-)live cells in green, percentage of live cells indicated.
(TIF)

S3 Fig BALF from patients with high inflammatory ARDS induces TLR9 signaling that can be blocked with TLR9 antagonists. BALF collected from patients with ARDS at day 1 of presentation and healthy were co-incubated with HEK-Blue hTLR9 reported cell line either alone (**A)** or with TLR9 antagonists ODN TTAGGG (**B**). Patients with ARDS were categorized as “highly inflammatory” (high PMN) if they contained > 80% neutrophils and > 2 mg/mL proteins or “low inflammatory” (low PMN) if they did not meet those criteria. After 24 hours BALF were analyzed for activity by spectroscopy of the target transgene NF-κB induced secreted embryonic alkaline phosphatase absorbance at 655 nm. Data are expressed as fold change over healthy volunteers (**A**) or in arbitrary units (**B**) and represent median with interquartile range of at least three independent experiments with the exception of the group of healthy volunteers (n = 2). *P < 0.05, Mann-Whitney test (**B**). CTL: controls (healthy volunteers), PMN: polymorphonuclear, ARDS: acute respiratory distress syndrome. A.U: arbitrary units.
(EPS)

S1 File Supplementary method. (DOCX)

## References

[1] Pugin J. Is the ventilator responsible for lung and systemic inflammation? Intensive Care Med. 2002;28(7):817–9. 10.1007/s00134-002-1320-8 . 12349817

[2] Tremblay L, Valenza F, Ribeiro SP, Li J, Slutsky AS. Injurious ventilatory strategies increase cytokines and c-fos m-RNA expression in an isolated rat lung model. J Clin Invest. 1997;99(5):944–52. 10.1172/JCI119259 9062352 PMC507902

[3] Ventilation with lower tidal volumes as compared with traditional tidal volumes for acute lung injury and the acute respiratory distress syndrome. The Acute Respiratory Distress Syndrome Network. N Engl J Med. 2000;342(18):1301–8. 10.1056/NEJM200005043421801 . 10793162

[4] Serpa Neto A, Cardoso SO, Manetta JA, Pereira VG, Esposito DC, Pasqualucci Mde O, et al Association between use of lung-protective ventilation with lower tidal volumes and clinical outcomes among patients without acute respiratory distress syndrome: a meta-analysis. JAMA. 2012;308(16):1651–9. 10.1001/jama.2012.13730 . 23093163

[5] Kawano T, Mori S, Cybulsky M, Burger R, Ballin A, Cutz E, et al Effect of granulocyte depletion in a ventilated surfactant-depleted lung. J Appl Physiol. 1987;62(1):27–33. 10.1152/jappl.1987.62.1.27 . 3558186

[6] Pugin J, Ricou B, Steinberg KP, Suter PM, Martin TR. Proinflammatory activity in bronchoalveolar lavage fluids from patients with ARDS, a prominent role for interleukin-1. Am J Respir Crit Care Med. 1996;153(6 Pt 1):1850–6. 10.1164/ajrccm.153.6.8665045 . 8665045

[7] Pugin J, Verghese G, Widmer MC, Matthay MA. The alveolar space is the site of intense inflammatory and profibrotic reactions in the early phase of acute respiratory distress syndrome. Crit Care Med. 1999;27(2):304–12. 10.1097/00003246-199902000-00036 . 10075054

[8] Dunn I, Pugin J. Mechanical ventilation of various human lung cells in vitro: identification of the macrophage as the main producer of inflammatory mediators. Chest. 1999;116(1 Suppl):95S–7S. 10.1378/chest.116.suppl_1.95s . 10424615

[9] Pugin J, Dunn I, Jolliet P, Tassaux D, Magnenat JL, Nicod LP, et al Activation of human macrophages by mechanical ventilation in vitro. Am J Physiol. 1998;275(6 Pt 1):L1040–50. 10.1152/ajplung.1998.275.6.L1040 . 9843840

[10] Bregeon F, Roch A, Delpierre S, Ghigo E, Autillo-Touati A, Kajikawa O, et al Conventional mechanical ventilation of healthy lungs induced pro-inflammatory cytokine gene transcription. Respir Physiol Neurobiol. 2002;132(2):191–203. 10.1016/s1569-9048(02)00069-1 . 12161332

[11] Charles PE, Martin L, Etienne M, Croisier D, Piroth L, Lequeu C, et al Influence of positive end-expiratory pressure (PEEP) on histopathological and bacteriological aspects of pneumonia during low tidal volume mechanical ventilation. Intensive Care Med. 2004;30(12):2263–70. 10.1007/s00134-004-2442-y . 15536527 PMC7095170

[12] Vaneker M, Halbertsma FJ, van Egmond J, Netea MG, Dijkman HB, Snijdelaar DG, et al Mechanical ventilation in healthy mice induces reversible pulmonary and systemic cytokine elevation with preserved alveolar integrity: an in vivo model using clinical relevant ventilation settings. Anesthesiology. 2007;107(3):419–26. 10.1097/01.anes.0000278908.22686.01 . 17721244

[13] Oudin S, Pugin J. Role of MAP kinase activation in interleukin-8 production by human BEAS-2B bronchial epithelial cells submitted to cyclic stretch. Am J Respir Cell Mol Biol. 2002;27(1):107–14. 10.1165/ajrcmb.27.1.4766 . 12091253

[14] Stroetz RW, Vlahakis NE, Walters BJ, Schroeder MA, Hubmayr RD. Validation of a new live cell strain system: characterization of plasma membrane stress failure. J Appl Physiol. 2001;90(6):2361–70. 10.1152/jappl.2001.90.6.2361 . 11356803

[15] Vlahakis NE, Hubmayr RD. Invited review: plasma membrane stress failure in alveolar epithelial cells. J Appl Physiol. 2000;89(6):2490–6;discussion 7. 10.1152/jappl.2000.89.6.2490 . 11090606

[16] McDonald B, Pittman K, Menezes GB, Hirota SA, Slaba I, Waterhouse CC, et al Intravascular danger signals guide neutrophils to sites of sterile inflammation. Science. 2010;330(6002):362–6. 10.1126/science.1195491 . 20947763

[17] Zhang Q, Raoof M, Chen Y, Sumi Y, Sursal T, Junger W, et al Circulating mitochondrial DAMPs cause inflammatory responses to injury. Nature. 2010;464(7285):104–7. 10.1038/nature08780 20203610 PMC2843437

[18] Grazioli S, Pugin J. Mitochondrial Damage-Associated Molecular Patterns: From Inflammatory Signaling to Human Diseases. Front Immunol. 2018;9:832 10.3389/fimmu.2018.00832 29780380 PMC5946030

[19] Kuipers MT, van der Poll T, Schultz MJ, Wieland CW. Bench-to-bedside review: Damage-associated molecular patterns in the onset of ventilator-induced lung injury. Crit Care. 2011;15(6):235 10.1186/cc10437 22216838 PMC3388678

[20] Matsuyama H, Amaya F, Hashimoto S, Ueno H, Beppu S, Mizuta M, et al Acute lung inflammation and ventilator-induced lung injury caused by ATP via the P2Y receptors: an experimental study. Respir Res. 2008;9:79 10.1186/1465-9921-9-79 19077288 PMC2627837

[21] Pugin J. How tissue injury alarms the immune system and causes a systemic inflammatory response syndrome. Ann Intensive Care. 2012;2(1):27 10.1186/2110-5820-2-27 22788849 PMC3488542

[22] Wu J, Yan Z, Schwartz DE, Yu J, Malik AB, Hu G. Activation of NLRP3 inflammasome in alveolar macrophages contributes to mechanical stretch-induced lung inflammation and injury. J Immunol. 2013;190(7):3590–9. 10.4049/jimmunol.1200860 23436933 PMC3608749

[23] Martinon F, Burns K, Tschopp J. The inflammasome: a molecular platform triggering activation of inflammatory caspases and processing of proIL-beta. Mol Cell. 2002;10(2):417–26. 10.1016/s1097-2765(02)00599-3 . 12191486

[24] Frank JA, Pittet JF, Wray C, Matthay MA. Protection from experimental ventilator-induced acute lung injury by IL-1 receptor blockade. Thorax. 2008;63(2):147–53. 10.1136/thx.2007.079608 . 17901159

[25] Charles PE, Tissieres P, Barbar SD, Croisier D, Dufour J, Dunn-Siegrist I, et al Mild-stretch mechanical ventilation upregulates toll-like receptor 2 and sensitizes the lung to bacterial lipopeptide. Crit Care. 2011;15(4):R181 10.1186/cc10330 21794115 PMC3387624

[26] Mortaz E, Adcock IM, Ito K, Kraneveld AD, Nijkamp FP, Folkerts G. Cigarette smoke induces CXCL8 production by human neutrophils via activation of TLR9 receptor. Eur Respir J. 2010;36(5):1143–54. 10.1183/09031936.00062209 . 19840968

[27] Panigrahi S, Ma Y, Hong L, Gao D, West XZ, Salomon RG, et al Engagement of platelet toll-like receptor 9 by novel endogenous ligands promotes platelet hyperreactivity and thrombosis. Circ Res. 2013;112(1):103–12. 10.1161/CIRCRESAHA.112.274241 23071157 PMC3537845

[28] Drifte G, Dunn-Siegrist I, Tissieres P, Pugin J. Innate immune functions of immature neutrophils in patients with sepsis and severe systemic inflammatory response syndrome. Crit Care Med. 2013;41(3):820–32. 10.1097/CCM.0b013e318274647d . 23348516

[29] Park WY, Goodman RB, Steinberg KP, Ruzinski JT, Radella F 2nd, Park DR, et al Cytokine balance in the lungs of patients with acute respiratory distress syndrome. Am J Respir Crit Care Med. 2001;164(10 Pt 1):1896–903. 10.1164/ajrccm.164.10.2104013 . 11734443

[30] Rich PB, Douillet CD, Mahler SA, Husain SA, Boucher RC. Adenosine triphosphate is released during injurious mechanical ventilation and contributes to lung edema. J Trauma. 2003;55(2):290–7. 10.1097/01.TA.0000078882.11919.AF . 12913640

[31] Ware LB, Matthay MA. The acute respiratory distress syndrome. N Engl J Med. 2000;342(18):1334–49. 10.1056/NEJM200005043421806 . 10793167

[32] Ranieri VM, Suter PM, Tortorella C, De Tullio R, Dayer JM, Brienza A, et al Effect of mechanical ventilation on inflammatory mediators in patients with acute respiratory distress syndrome: a randomized controlled trial. JAMA. 1999;282(1):54–61. 10.1001/jama.282.1.54 . 10404912

[33] Tschopp J. Mitochondria: Sovereign of inflammation? Eur J Immunol. 2011;41(5):1196–202. 10.1002/eji.201141436 . 21469137

[34] Zhang JZ, Liu Z, Liu J, Ren JX, Sun TS. Mitochondrial DNA induces inflammation and increases TLR9/NF-kappaB expression in lung tissue. Int J Mol Med. 2014;33(4):817–24. 10.3892/ijmm.2014.1650 . 24535292 PMC3976143

[35] McIlroy DJ, Jarnicki AG, Au GG, Lott N, Smith DW, Hansbro PM, et al Mitochondrial DNA neutrophil extracellular traps are formed after trauma and subsequent surgery. J Crit Care. 2014;29(6):1133 e1-5. 10.1016/j.jcrc.2014.07.013 . 25128442

[36] McIlroy DJ, Bigland M, White AE, Hardy BM, Lott N, Smith DW, et al Cell necrosis-independent sustained mitochondrial and nuclear DNA release following trauma surgery. J Trauma Acute Care Surg. 2015;78(2):282–8. 10.1097/TA.0000000000000519 25602756 PMC4323572

[37] Yao X, Carlson D, Sun Y, Ma L, Wolf SE, Minei JP, et al Mitochondrial ROS Induces Cardiac Inflammation via a Pathway through mtDNA Damage in a Pneumonia-Related Sepsis Model. PLoS One. 2015;10(10):e0139416 10.1371/journal.pone.0139416 26448624 PMC4598156

[38] Unuma K, Aki T, Funakoshi T, Hashimoto K, Uemura K. Extrusion of mitochondrial contents from lipopolysaccharide-stimulated cells: Involvement of autophagy. Autophagy. 2015;11(9):1520–36. 10.1080/15548627.2015.1063765 26102061 PMC4590602

[39] McLelland GL, Soubannier V, Chen CX, McBride HM, Fon EA. Parkin and PINK1 function in a vesicular trafficking pathway regulating mitochondrial quality control. EMBO J. 2014;33(4):282–95. 10.1002/embj.201385902 24446486 PMC3989637

[40] Ali MH, Pearlstein DP, Mathieu CE, Schumacker PT. Mitochondrial requirement for endothelial responses to cyclic strain: implications for mechanotransduction. Am J Physiol Lung Cell Mol Physiol. 2004;287(3):L486–96. 10.1152/ajplung.00389.2003 . 15090367

[41] Davidson BA, Vethanayagam RR, Grimm MJ, Mullan BA, Raghavendran K, Blackwell TS, et al NADPH oxidase and Nrf2 regulate gastric aspiration-induced inflammation and acute lung injury. J Immunol. 2013;190(4):1714–24. 10.4049/jimmunol.1202410 23296708 PMC3563868

[42] Lin JY, Jing R, Lin F, Ge WY, Dai HJ, Pan L. High Tidal Volume Induces Mitochondria Damage and Releases Mitochondrial DNA to Aggravate the Ventilator-Induced Lung Injury. Front Immunol. 2018;9:1477 10.3389/fimmu.2018.01477 30018615 PMC6037891

[43] Timmermans K, Kox M, Vaneker M, Pickkers P, Scheffer GJ. Mitochondrial DNA and TLR9 Signaling Is Not Involved in Mechanical Ventilation-Induced Inflammation. Anesth Analg. 2017;124(2):531–4. 10.1213/ANE.0000000000001554 28099322

[44] Blot M, Pauchard LA, Dunn I, Donze J, Malnuit S, Rebaud C, et al Mechanical ventilation and Streptococcus pneumoniae pneumonia alter mitochondrial homeostasis. Sci Rep. 2018;8(1):11718 10.1038/s41598-018-30226-x 30082877 PMC6078986

[45] Haden DW, Suliman HB, Carraway MS, Welty-Wolf KE, Ali AS, Shitara H, et al Mitochondrial biogenesis restores oxidative metabolism during Staphylococcus aureus sepsis. Am J Respir Crit Care Med. 2007;176(8):768–77. 10.1164/rccm.200701-161OC 17600279 PMC2020830

[46] Hajizadeh S, DeGroot J, TeKoppele JM, Tarkowski A, Collins LV. Extracellular mitochondrial DNA and oxidatively damaged DNA in synovial fluid of patients with rheumatoid arthritis. Arthritis Res Ther. 2003;5(5):R234–40. 10.1186/ar787 12932286 PMC193725

[47] Sarma JV, Ward PA. Oxidants and redox signaling in acute lung injury. Compr Physiol. 2011;1(3):1365–81. 10.1002/cphy.c100068 . 23733646

[48] Abraham E. Neutrophils and acute lung injury. Crit Care Med. 2003;31(4 Suppl):S195–9. 10.1097/01.CCM.0000057843.47705.E8 . 12682440

[49] Crouser ED, Shao G, Julian MW, Macre JE, Shadel GS, Tridandapani S, et al Monocyte activation by necrotic cells is promoted by mitochondrial proteins and formyl peptide receptors. Crit Care Med. 2009;37(6):2000–9. 10.1097/CCM.0b013e3181a001ae 19384205 PMC2743203

[50] Narimanbekov IO, Rozycki HJ. Effect of IL-1 blockade on inflammatory manifestations of acute ventilator-induced lung injury in a rabbit model. Exp Lung Res. 1995;21(2):239–54. 10.3109/01902149509068830 . 7774527

[51] Gross O, Thomas CJ, Guarda G, Tschopp J. The inflammasome: an integrated view. Immunol Rev. 2011;243(1):136–51. 10.1111/j.1600-065X.2011.01046.x . 21884173

[52] Kuipers MT, Aslami H, Janczy JR, van der Sluijs KF, Vlaar AP, Wolthuis EK, et al Ventilator-induced lung injury is mediated by the NLRP3 inflammasome. Anesthesiology. 2012;116(5):1104–15. 10.1097/ALN.0b013e3182518bc0 22531249

[53] Nakahira K, Kyung SY, Rogers AJ, Gazourian L, Youn S, Massaro AF, et al Circulating mitochondrial DNA in patients in the ICU as a marker of mortality: derivation and validation. PLoS Med. 2013;10(12):e1001577; discussion e. 10.1371/journal.pmed.1001577 24391478 PMC3876981

[54] Matzinger P. Tolerance, danger, and the extended family. Annu Rev Immunol. 1994;12:991–1045. 10.1146/annurev.iy.12.040194.005015 . 8011301

[55] Matzinger P. Friendly and dangerous signals: is the tissue in control? Nat Immunol. 2007;8(1):11–3. 10.1038/ni0107-11 . 17179963

[56] Bregeon F, Delpierre S, Chetaille B, Kajikawa O, Martin TR, Autillo-Touati A, et al Mechanical ventilation affects lung function and cytokine production in an experimental model of endotoxemia. Anesthesiology. 2005;102(2):331–9. 10.1097/00000542-200502000-00015 . 15681948

