# Supplementary figures and images for: Mitochondrial alarmins are tissue mediators of ventilator-induced lung injury and ARDS
Source: PLoS One. 2019 Nov 22;14(11):e0225468. doi: 10.1371/journal.pone.0225468 (PMC6874419; doi:10.1371/journal.pone.0225468)

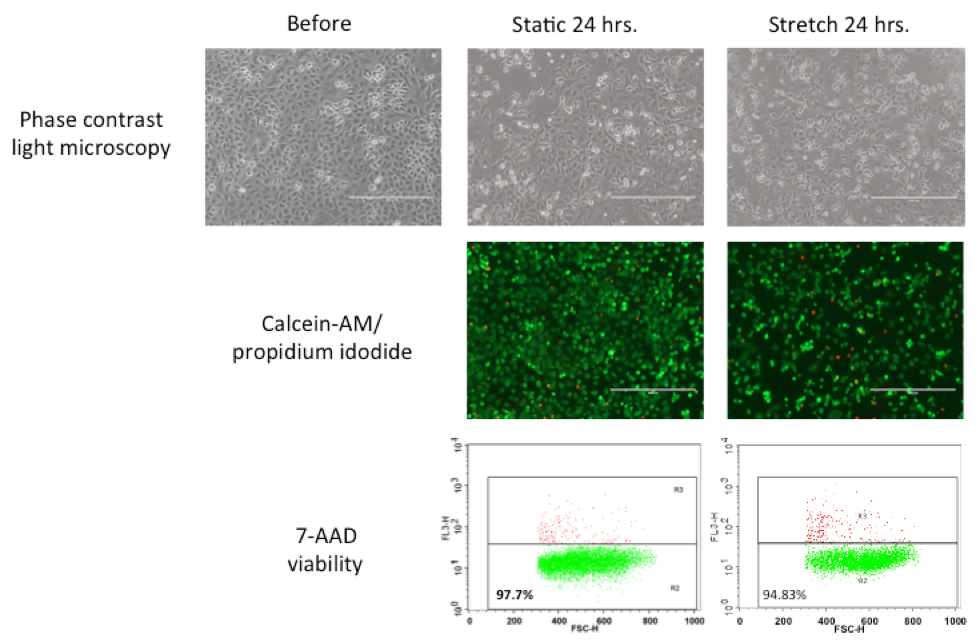

Supplement: S2 Fig — A549 cells viability after 24 hrs of cell stretching. Upper panels, phase contrast microscopy; middle panels, fluorescence microscopy (calcein(+)live cells in green, and propidium iodide(+)nuclei of dead cells in red); lower panels, flow cytometry analysis of stretched vs. static cells; 7-AAD(+)dead cells in red, 7-AAD(-)live cells in green, percentage of live cells indicated. (TIF) [file pone.0225468.s002.tif]
