# Supplementary material for: Mitochondrial alarmins are tissue mediators of ventilator-induced lung injury and ARDS
Source: PLoS One. 2019 Nov 22;14(11):e0225468. doi: 10.1371/journal.pone.0225468 (PMC6874419; doi:10.1371/journal.pone.0225468)
Supplement: S1 File — (DOCX) [file pone.0225468.s004.docx]

**Online data supplement**

**Material and Methods**

**Cell stretching**

A549 cells were cultured in Bioflex® plates up to 80% confluence in DMEM/F12 Glutamax® medium (Invitrogen, Basel, Switzerland) containing 10% fetal bovine serum (Gibco, Basel, Switzerland) and antibiotics. Cells were then submitted to cyclic stretch at a stretching rate of 20 cycles/minute with a square signal, a 1:1 stretch:relaxation ratio, and a 20% maximal equibiaxial elongation for 24 to 48 hrs., in a 37°C incubator with 5% CO_2_. Supernatants from stretched cells and from cells grown in the same conditions in Bioflex® plates without cell stretching were sampled at different times, and used for the quantification of alarmins, as well as for other functional tests.

**Measurement of alarmins in conditioned supernatants**

**Mitochondrial DNA.** DNA was isolated using the DNA purification kit from blood or body fluids (Qiagen, Valencia, CA, USA), with a final volume of 200 µl of DNA resuspended in elution buffer. Quantitative PCR using the IQ^TM^ Sybr Green technique (BioRad, Hercules, CA, USA) was performed with one-tenth and one-hundredth dilutions of the final product, compared to a standard curve of human mitochondrial DNA to quantify the amount of mitochondrial DNA amplified, and expressed as arbitrary units. Melting curves were performed to ascertain the amplification of a single amplicon. Human mitochondrial DNA was isolated from A459 cells using the mitochondrial isolation kit for cultured cells from ThermoScientific (Rockford, IL, USA).

**Chemotactic factors.** Human neutrophils from healthy volunteers were isolated using a Ficoll-Paque^TM^ (GE Healthcare Bio-Scienses AB, Uppsala, Sweden) gradient and seeded into the upper well. Supernatants from cells stretched for various times were put into the lower well. Results were expressed as % of neutrophils from the upper well having migrated across the filter to the lower well after 90 min. Neutrophils in the lower well were detected with the DraQ5^TM^ dye (Biostatus) marking neutrophil DNA, counted using the Applied Biosystems 8200 Cellular Detection System (Life Technologies, Switzerland), and a known concentration of neutrophils as standard.

**Mechanical ventilation in rabbits**

**Lung mediators and lung injury score.** Tissue IL-8, IL-1ß and TNF-α protein levels were quantified using rabbit specific ELISA kits following the manufacturer’s instructions (Euromedex, Strasbourg, France). Blood samples were obtained before intubation, 4 and 8 hrs. after intubation. Plasma levels of IL-8, IL-1ß and TNF-α were measured using the same ELISA kits. For ultrastructural examination, approximately 1 cm^3^ of tissue was fixed in formalin and embedded in paraffin. Four-μm sections were obtained and stained with hematoxylin-eosin. A pulmonary pathologist blinded to the treatment group examined ten fields of each section and an injury score was calculated as previously described ([28](#_ENREF_28)). Briefly, lung injury assessment was based on the degree of neutrophil infiltration, haemorrhage and edema. Lung injury was considered absent [0], mild [1], moderate [2] or severe [3]. Another lung sample was harvested for RNA extraction using the GenElute kit (Sigma, Dorset, UK) according to the manufacturer’s instructions. Complementary DNA (cDNA) was obtained by reverse transcription using random primers, RNAsin treatment, and ImProm II reverse transcriptase (Promega, Madison, WI). Quantitative PCR was performed using the IQ5 thermocycler (Biorad, Hercules, CA) and the IQ^TM^ Syber Green Supermix (Biorad) and rabbit-specific primers, designed using the Primer3 software (version 0.4.0), and the rabbit (*Oryctolagus cuniculus*) sequence database. Melting curves were performed to ascertain the specificity of the amplification. The following primers were used: *rGapdh* forward: 5’-ATG TTT GTG ATG GGC GTG AAC C-3’, reverse: 5’-CCC AGC ATC GAA GGT AGA GGA-3’; *rIl-8* forward: 5’-AAC CTT CCT GCT GCT TCT GA-3’, reverse: 5’-TCT GCA CCC ACT TTT TCC TTG-3’. The results were expressed as expression levels normalized to a reference gene (*rGapdh*).

**Mitochondrial alarmins in rabbit BAL fluids.** Quantitative PCR was used to measure levels of mitochondrial DNA in BAL fluid (cytochrome B, cytochrome C oxidase III, and NADH I). BAL fluid DNA was isolated using the DNA purification kit from blood or body fluids (Qiagen) using 200 µl of sample. Rabbit primer sequences were synthesized by Microsynth (Balgach, Switzerland) and had no significant homology with sequences from rabbit genomic DNA (Blast® site, http://blast.ncbi.nlm.nih.gov). The following primers were used: rabbit cytochrome B, forward 5’-CCA TCC TTG TTC TAG CCT TCA and reverse 5’-AAT GGT GAT GAA CGG GTG TT; rabbit cytochrome C oxidase III, forward 5'-GAA GGC AAT CGC AAA AAC AT and reverse 5'-ACG TGA AGA CCG TGA AAT CC); NADH I, forward 5' GCC CCA ACC CTA GCT CTA AC and reverse 5'-GCT CGG AGA GCA CCA AAT AG. Quantitative PCR was used to measure levels of mitochondrial DNA in BAL fluid using the IQ^TM^ Sybr Green technique (Biorad), and quantified using a standard curve of rabbit mitochondrial DNA. Rabbit mitochondrial DNA was isolated from rabbit peripheral blood mononuclear cells obtained from fresh rabbit heparinized blood and a Ficoll-Paque^TM^ gradient, using the mitochondrial isolation kit for cultured cells from ThermoScientific. The DNA concentration was measured by spectrometry (Nanodrop ND-1000) and resuspended at the stock concentration of 1 µg/ml. Levels of rabbit BAL fluid ATP was measured using the same technology as for human samples.

**Chemotactic activity of rabbit BAL fluid** was also measured according to the protocol described above for supernatants from human stretched cells, using human neutrophils.
